# Nomogram Analysis for Predicting Response to Androgen‐Receptor‐Axis‐Targeted Therapies in Patients With Metastatic Castration‐Resistant Prostate Cancer

**DOI:** 10.1002/cam4.70319

**Published:** 2024-11-08

**Authors:** I‐Hung Shao, Hsiang‐Shen Wang, Chin‐Hsuan Hsieh, Tsung‐Lin Lee, Ying‐Hsu Chang, Liang‐Kang Huang, Yuan‐Cheng Chu, Hung‐Chen Kan, Po‐Hung Lin, Kai‐Jie Yu, Chun‐Te Wu, Cheng‐Keng Chuang, See‐Tong Pang

**Affiliations:** ^1^ Department of Surgery, Division of Urology Chang Gung Memorial Hospital, Linkou Branch Taoyuan Taiwan; ^2^ School of Medicine, College of Medicine Chang Gung University Taoyuan Taiwan; ^3^ Department of Pathology Chang Gung Memorial Hospital, Linkou Branch Taoyuan Taiwan; ^4^ Department of Surgery, Division of Urology New Taipei Municipal TuCheng Hospital New Taipei Taiwan

**Keywords:** androgen deprivation therapy (ADT), androgen‐receptor‐axis‐targeted therapies (ARAT), metastatic castration‐resistant prostate cancer (mCRPC), nomogram analysis, prostate cancer, prostate‐specific antigen (PSA)

## Abstract

**Background:**

This study aimed to identify the clinical predictors for the response of patients with mCRPC to ARATs.

**Materials and Methods:**

We retrospectively collected data on consecutive patients who were diagnosed with mCRPC and underwent ARAT treatment during this stage of the disease. Clinical parameters were obtained through medical chart reviews. ARAT failure was defined as a continuous increase in the serum prostate‐specific antigen (PSA) level above nadir to > 2 ng/mL, accompanied by radiographic progression. ARAT failure‐free survival and overall survival were assessed through Kaplan–Meier survival analysis and Cox regression survival analysis. Nomogram analysis based on significant predictors of ARAT failure‐free survival was performed.

**Results:**

In total, 319 patients with mCRPC who underwent ARAT were included. Multivariate analysis revealed that age, International Society of Urological Pathology (ISUP) grading, and chemotherapy‐naïve status were significant predictors of ARAT failure‐free survival. For overall survival, age, ISUP grading, and nadir PSA level during androgen deprivation therapy (ADT) were significant predictors. Through nomogram analysis based on age, ISUP grading, and chemotherapy‐naïve status, the likelihood of ARAT duration being more or less than 1 year could be predicted.

**Conclusion:**

For mCRPC patients, being older, having ISUP Grade 5 cancer, and having a history of chemotherapy were associated with a shorter duration of response to next‐line ARATs. Therefore, other therapeutic agents should be prioritized for such patients. Notably, among the included patients, those who were older, had a higher ISUP grade and a higher nadir PSA level during ADT exhibited worse overall survival.

## 1 Introduction

Prostate cancer is the second most common male malignancy worldwide; in the United States, more than 200,000 newly diagnosed prostate cancer cases and 30,000 deaths from prostate cancer are reported annually, and this trend is increasing [1]. The prognosis of prostate cancer varies depending on the disease stage. For the localized regional stage, the 5‐year survival rate is > 95%. In this stage, the primary treatment modalities include radical surgery, radiation therapy, and ablation therapy. For metastatic prostate cancer, which accounts for approximately 30% of newly diagnosed prostate cancer cases, the 5‐year survival rate is only 30%, which is a key factor influencing prostate‐cancer‐specific survival [2, 3].

Androgen deprivation therapy (ADT) is the mainstay treatment for metastatic prostate cancer because of both benign prostate tissue and malignant prostate tissue express androgen receptor (AR), which prostate cancer cells require for their growth and cancer progression [4, 5].

However, even when adequate ADT is provided, prostate cancer almost always relapses and progresses to the subsequent stage, that is, castration‐resistant prostate cancer (CRPC) [6, 7]. With the progression of prostate cancer to CRPC, the median overall survival (OS) is 19–30 months according to real‐world data [8, 9, 10]. The mechanism of ADT resistance includes mutation, amplification, and crosstalk between the cellular signal pathways related to the androgen receptor (AR) gene, and the androgen autocrine system of cancer cells [11].

To overcome resistance to ADT alone, multiple therapy agents have been identified, including a new generation of androgen‐receptor‐axis‐targeted therapies (ARATs), which has been demonstrated to be an effective first‐line therapy for prolonging the survival of patients with mCRPC [12, 13, 14]. Prescribed orally, ARATs include abiraterone acetate, a CYP 17 inhibitor that blocks androgen synthesis [15]; and enzalutamide, apalutamide, and darolutamide, which are androgen receptor antagonists that competitively suppress androgen‐induced AR activation [16, 17].

In addition to ARAT, other treatments have been demonstrated to exhibit high efficacy in patients with mCRPC. To date, the standard treatments for mCRPC include chemotherapy (docetaxel and cabazitaxel), poly ADP‐ribose polymerase (PARP) inhibitors (olaparib and rucaparib), radiopharmaceutical therapies (radium‐223 and Lutetium‐177–PSMA‐617), and two immunotherapy treatments (sipuleucel‐T and pembrolizumab) [18, 19, 20, 21, 22, 23, 24, 25, 26, 27].

Although mCRPC is the most lethal and terminal stage of prostate cancer, multiple treatment modalities are still available at this stage, and different treatment agents are associated with varying responses and durations of effectiveness. When a patient with prostate cancer develops drug resistance, next‐line therapy must be started as soon as possible. Furthermore, drugs may exhibit cross‐resistance; thus, the sequence of treatments is crucial for improving the prognosis of mCRPC. For this reason, how to predict the response of each agent is a key clinical need that must be met. Because of their oral route of administration, ease of use, and minimal side effects, ARATs are commonly used as first‐line treatment for mCRPC. However, a reliable method for predicting the response of patients with mCRPC to first‐line ARAT has yet to be established. In the present study, we aimed to discover the predictors of the response to ARAT in patients with mCRPC.

## 2 Materials and Methods

### 2.1 Patients

We conducted a retrospective study that included consecutive patients who were diagnosed with metastatic prostate cancer and underwent ARAT treatment between June 1999 and November 2020 in one of four tertiary medical centers.

We reviewed the medical charts of the included patients who fulfilled the criteria for CRPC, which was either clinical or biochemical progression with a serum testosterone concentration of < 50 ng/dL in an adequate castrate environment. Clinical progression was defined on the basis of radiological imaging results and in accordance with the Response Evaluation Criteria in Solid Tumors (RECIST), whereas biochemical progression was defined as a consecutive increase in serum prostate‐specific antigen (PSA) levels by > 25% over two measurements taken at least 1‐week apart, with the absolute value being > 2 ng/mL. Patients who progressed to CRPC and underwent next‐generation hormonal agent therapy were included in the present study. ARAT failure was defined as a consecutive increase in serum PSA levels above nadir to > 2 ng/mL, accompanied by radiographic progression.

Patients with a history of any other active malignancy in the 5 years preceding the study period were excluded. All included patients were comprehensively evaluated, and the included cases were discussed during our urology–oncology multidisciplinary meeting. These patients were managed as per the clinical guidelines for prostate cancer.

The present study was approved by the Institutional Review Board (IRB) of Chang‐Gung Memorial Hospital. Data collection was performed as per the confidentiality requirements in the Declaration of Helsinki. Because of the retrospective design of the present study, the requirement to obtain consent from patients to review their medical records was waived by the aforementioned IRB.

### 2.2 Data Collection

Data pertaining to various preoperative general characteristics (i.e., sex age, body height, body weight, body mass index [BMI], and prostate volume) and tumor‐related parameters (e.g., initial PSA [iPSA] level, International Society of Urological Pathology [ISUP] grading, de novo metastatic status, ADT/ARAT agents, and serum marker–related parameters) were retrieved.

All patients underwent regular follow‐up evaluations and serum PSA testing at 3‐month intervals. Image surveys, including computed tomography scans and bone scans, were performed on the basis of clinical conditions.

### 2.3 Statistical Analyses

Cox regression analysis was performed to determine ARAT failure‐free survival and OS. Univariate analysis and, subsequently, multivariate analysis were performed for the parameters with a *p* < 0.05. ARAT failure‐free survival and OS were determined through Kaplan–Meier survival analysis, and Cox regression survival analysis was performed for parameters with significant results in both groups. For statistical analyses, a *p* < 0.05 was regarded as significant. All statistical analyses were performed using IBM SPSS Statistics version 22.0. Armonk, NY: IBM Corp. Nomogram analysis based on significant predictors of ARAT failure‐free survival was performed using the programming language R.

## 3 Results

After the screening was performed, 319 patients with mCRPC who underwent ARAT were included in the present study. The mean age of the patients at the time of diagnosis of prostate cancer was 70.1 years, and their general characteristics (e.g., body height, body weight, and BMI) are listed in Table 1.

**TABLE 1:** Patients' general characteristics.

Variables	Mean/number	SD	Range/percentage
Patients' general characteristics			
Total number	319		
Age	70.1	9.1	43–97 year‐old
Height	163.8	6.0	165–179.9 cm
Weight	66.8	10.8	43.1–155.3 kg
BMI	24.9	3.6	17.1–56.6 kg/cm^2^
Prostate volume			
Total	49.1	28.6	3.3–183 gm
T‐zone	20.7	15.8	0.6–72.4
Cancer related parameters			
iPSA	629.5	1834.6	2.6–23125.5 ng/mL
ISUP group			
1	15		5.1%
2	18		6.1%
3	27		9.1%
4	66		22.3%
5	170		57.4%
DeNovo distant mets			
0	65		20.4%
1	254		79.6%
ADT agent			
Degarelix	49		15.4%
Goserelin acetate	78		24.5%
Leuprorelin acetate	160		50.2%
Triptorelin	1		0.3%
Orchiectomy	29		9.1%
1st ARAT agent			
Abiraterone acetate	177		55.5%
Enzalutamide	142		44.5%
Pre‐ or post‐ Chemo setting			
Pre‐chemo	67		21.0%
Post‐chemo	85		26.6%
No chemo	167		52.4%
Nadir PSA during ADT	13.74	45.7	0–354.1 ng/mL
PSA when strating ARAT	214.05	606.9	0.008–5285.2 ng/mL
Follow‐up	79.53	50.6	11.7–247.2 months

Abbreviations: ADT = androgen deprivation therapy; ARAT = androgen receptor‐axis‐targeted therapies; BMI = Body Mass Index; ISUP = International Society of Urological Pathology; PSA = prostate‐specific antigen.

**FIGURE 1:**
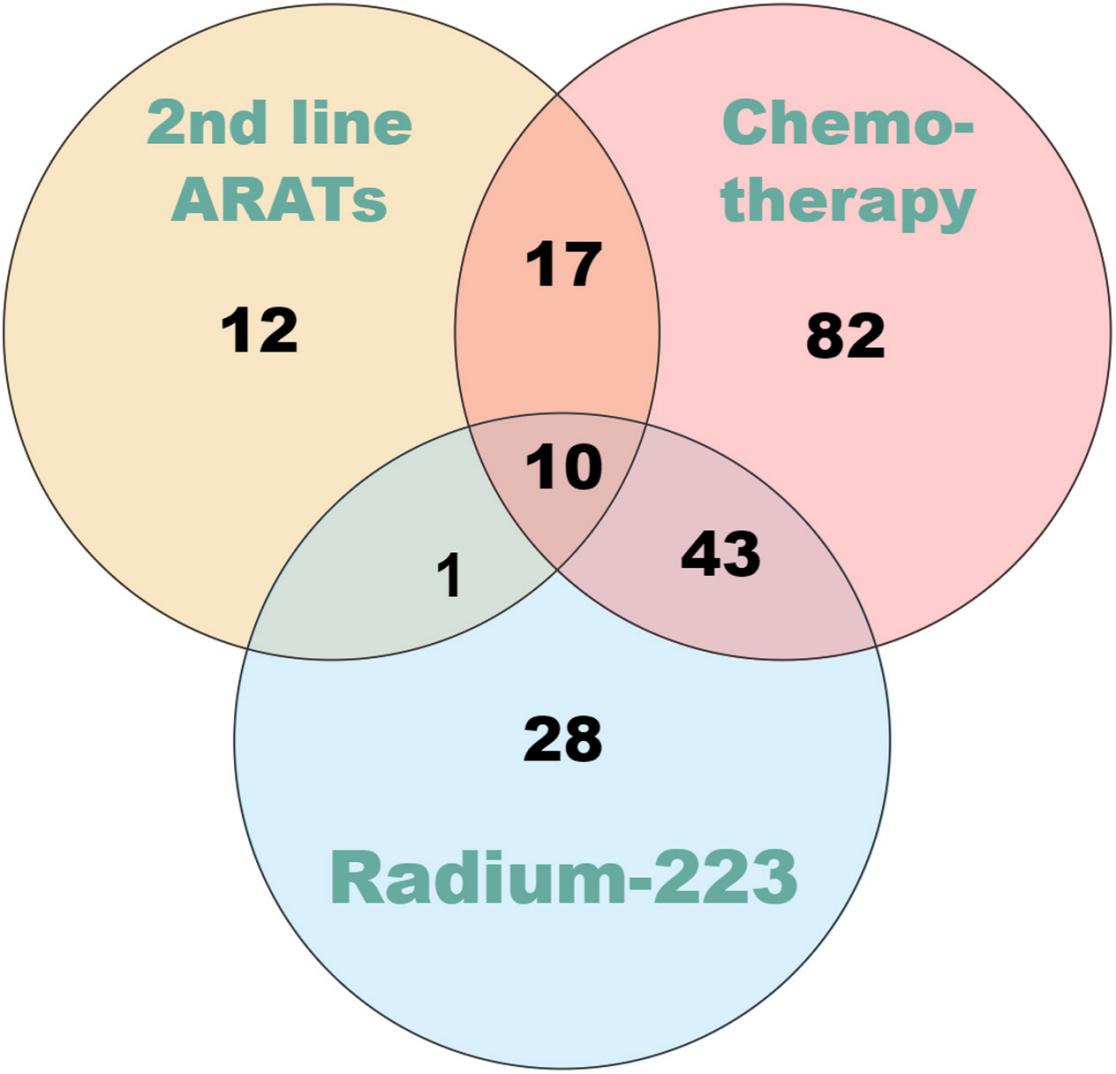
Life‐prolonged treatments after 1st ARATs in mCRPC stage.

The patients had a mean iPSA level of 629.5 ng/mL, and approximately 80% of them had ISUP Grades 4 and 5 cancer. Overall, 79.6% of the patients had de novo metastatic disease, whereas the remaining 20.4% were initially diagnosed with localized disease. After the patients progressed to mCRPC and started next‐line treatment, 55.5% underwent treatment with abiraterone acetate combined with prednisolone, whereas the remaining 44.5% underwent treatment with enzalutamide. The other cancer‐related data collected are listed in Table 1. In total, 193 (60.5%) patients underwent second‐line life‐prolonging treatment for mCRPC, including second‐line ARATs, chemotherapy, and radium‐223 treatment. Among these patients, 10 (3.1%) patients subsequently underwent third‐line treatment. The ratios pertaining to these findings are presented in Figure 1.

To determine ARAT failure‐free survival, we performed Cox regression to analyze the potential predictors of ARAT failure‐free survival, including various general characteristics and prostate cancer‐related parameters. Through univariate analysis, age, BMI, Eastern Cooperative Oncology Group performance status (ECOG‐PS), ISUP grading, first ARAT agent, chemotherapy‐naïve status, and nadir PSA during ADT were revealed to have significant effects on ARAT failure‐free survival. However, through multivariate analysis, significant results were only obtained for age (*p* = 0.021), ISUP grading (*p* = 0.001), and chemotherapy‐naïve status (*p* < 0.0001). A detailed breakdown of these analysis results is provided in Table 2.

**TABLE 2:** Cox‐regression uni‐variate analysis for ARAT‐failure survival.

	Univariate analysis						Multi‐variate analysis	
	B	SE	Wald	df	*p*	Exp (B)	*p*	Exp (B)
General parameters								
Age	0.013	0.006	4.454	1.000	0.035*	1.013	0.021*	1.020
BMI	−0.033	0.017	3.890	1.000	0.049*	0.968	0.152	0.974
ECOG	−1.056	0.589	3.223	1.000	0.002**	0.348	0.471	
ISUP Group	0.154	0.055	7.787	1.000	0.005**	1.166	0.001**	1.223
DeNovo Mets	0.172	0.143	1.434	1.000	0.231	1.187		
1st ARAT agent	0.278	0.117	5.619	1.000	0.018*	1.321	0.386	1.128
Chemotherapy Naïve	−0.586	0.131	20.137	1.000	< 0.0001**	0.556	< 0.0001**	0.407
Duration from HSPC to CRPC	0.000	0.000	1.300	1.000	0.254	1.000		
Serum markers								
iPSA	0.000	0.000	0.725	1.000	0.395	1.000		
Nadir PSA during ADT	0.003	0.001	8.444	1.000	0.004**	1.003	0.421	1.001
PSA when starting ARAT	0.000	0.000	3.504	1.000	0.061	1.000		

Abbreviations: ADT = androgen deprivation therapy; ARAT = androgen receptor‐axis‐targeted therapies; BMI = Body Mass Index; ECOG = Eastern Cooperative Oncology Group; ISUP = International Society of Urological Pathology; PSA = prostate‐specific antigen.

* *p* < 0.05.

** *p* < 0.01.

For OS, our univariate analysis revealed that age, BMI, ISUP grading, and nadir PSA during ADT had significant effects on OS. Through multivariate analysis, significant results were only obtained for age (*p* = 0.029), ISUP grading (*p* = 0.012), and nadir PSA during ADT (*p* < 0.0001). A detailed breakdown of these analysis results is provided in Table 3.

**TABLE 3:** Cox‐regression uni‐variate analysis for overall survival.

	Univariate analysis						Multi‐variate analysis	
	B	SE	Wald	df	*p*	Exp (B)	*p*	Exp (B)
General parameters								
Age	0.025	0.008	9.722	1.000	0.002**	1.025	0.029*	1.020
BMI	−0.045	0.021	4.494	1.000	0.032*	0.956	0.079	0.963
ECOG			0.044	1.000	0.934	0.860		
ISUP Group	0.162	0.066	5.928	1.000	0.013*	1.176	0.012*	1.183
DeNovo Mets	−0.085	0.165	0.266	1.000	0.606	0.919		
1st ARAT agent	0.269	0.139	3.716	1.000	0.054	1.308		
Pre‐ or post‐chemotherapy ARAT								
Duration from HSPC to CRPC	0.000	0.000	2.671	1.000	0.102	1.000		
Serum markers								
iPSA	0.000	0.000	0.309	1.000	0.578	1.000		
Nadir PSA during ADT	0.005	0.001	18.221	1.000	0.00002**	1.005	0.00009**	1.005
PSA when starting ARAT	0.000	0.000	0.219	1.000	0.640	1.000		

Abbreviations: ADT = androgen deprivation therapy; ARAT = androgen receptor‐axis‐targeted therapies; BMI = Body Mass Index; ECOG = Eastern Cooperative Oncology Group; ISUP = International Society of Urological Pathology; PSA = prostate‐specific antigen.

* *p* < 0.05.

** *p* < 0.01.

**FIGURE 2:**
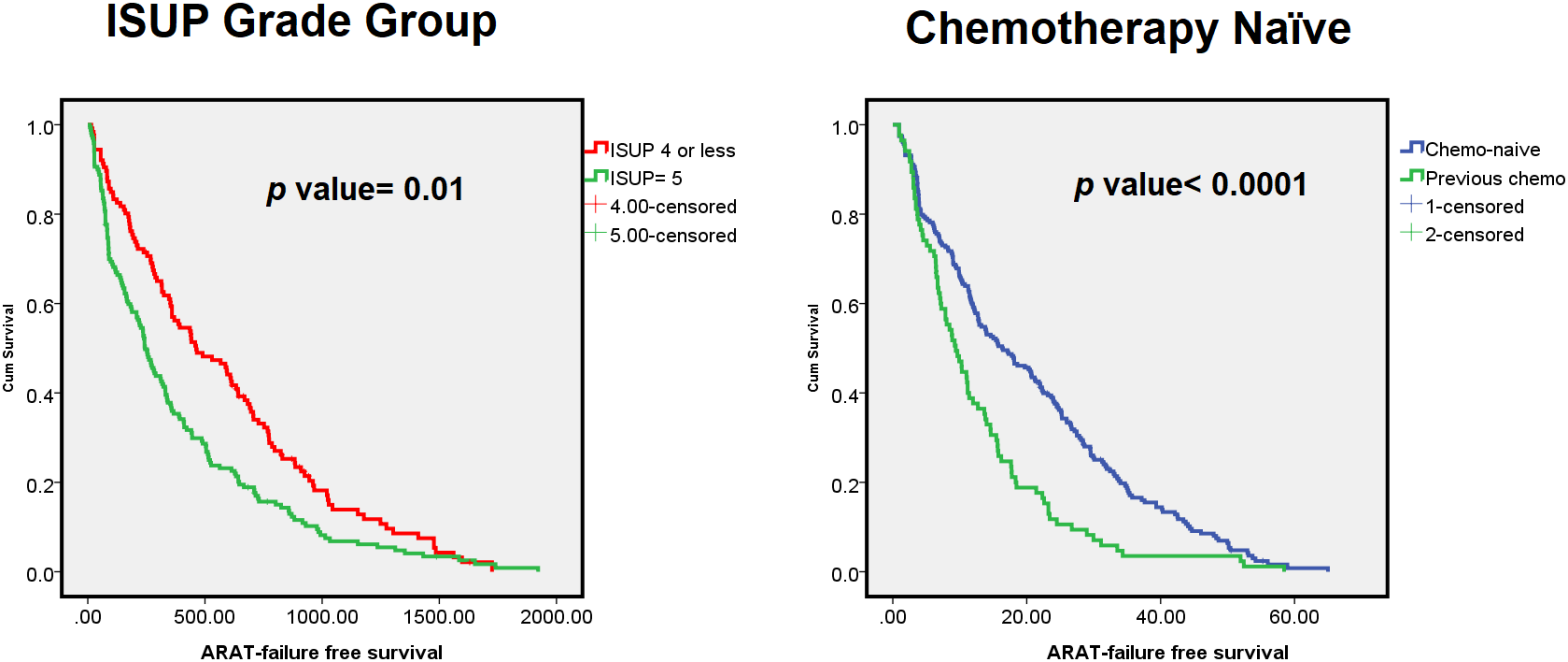
Kaplan–Meier survival plots for ARAT failure‐free survival. (left) Subgroup with ISUP grade group ≦ 4 or 5. (right) Subgroup with chemotherapy naïve or not.

**FIGURE 3:**
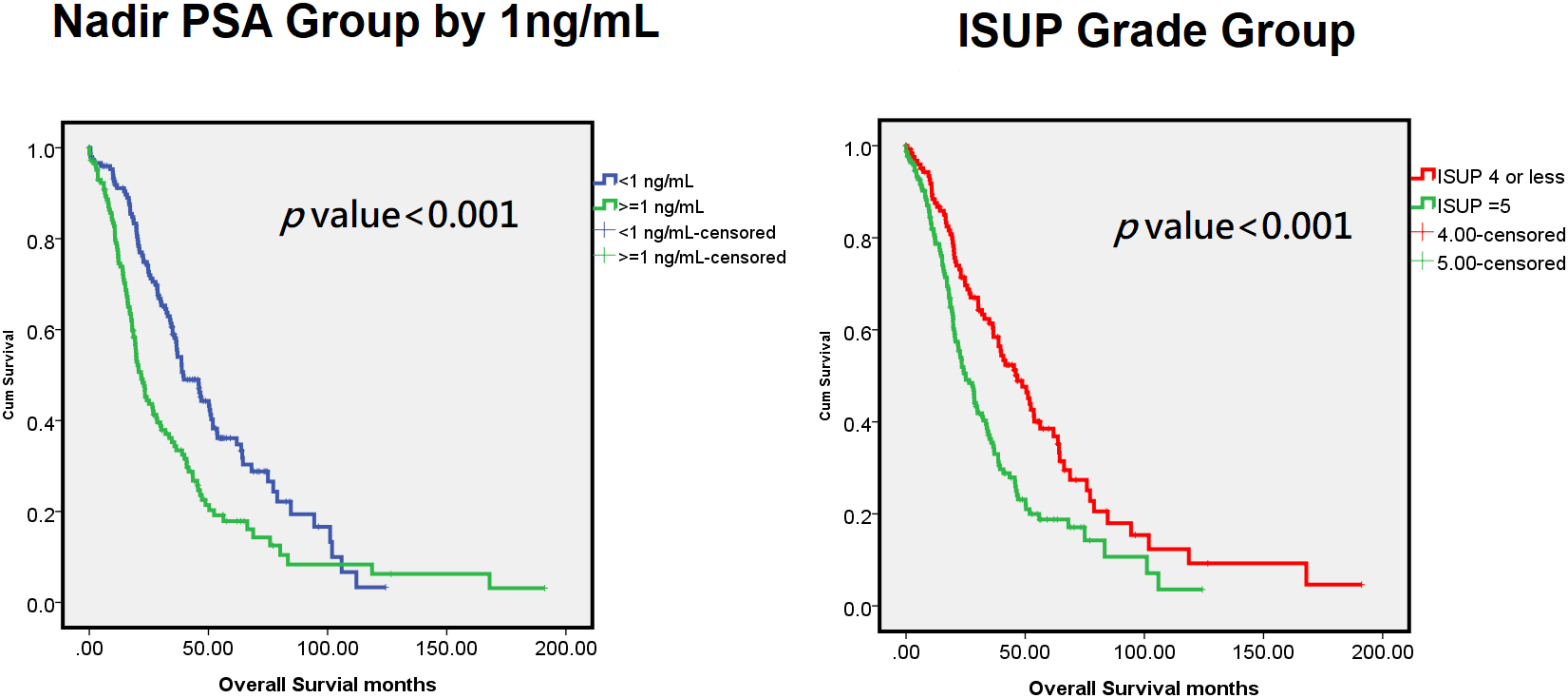
Kaplan–Meier survival plots for overall survival. (left) Subgroup with nadir PSA during ADT < 1 ng/mL and ≧ 1 ng/mL. (right) Subgroup with ISUP grade group ≦ 4 or 5.

Furthermore, a Kaplan–Meier survival curve was generated to analyze the differing effects of ISUP grading and chemotherapy‐naïve status on ARAT failure‐free survival. The curve revealed that the patients with ISUP Grade ≤ 4 cancer and those who were chemotherapy‐naïve achieved survival outcomes that were significantly more favorable than those achieved by the patients who underwent treatment with abiraterone (*p* = 0.01) and those who had previously undergone chemotherapy (*p* < 0.0001). Our OS analysis revealed significantly higher OS rates for the patients with a nadir PSA level of < 1 ng/mL during ADT (*p* < 0.001) and those with ISUP Grade ≤ 4 cancer (*p* < 0.001) (Figures 2 and 3).

**FIGURE 4:**
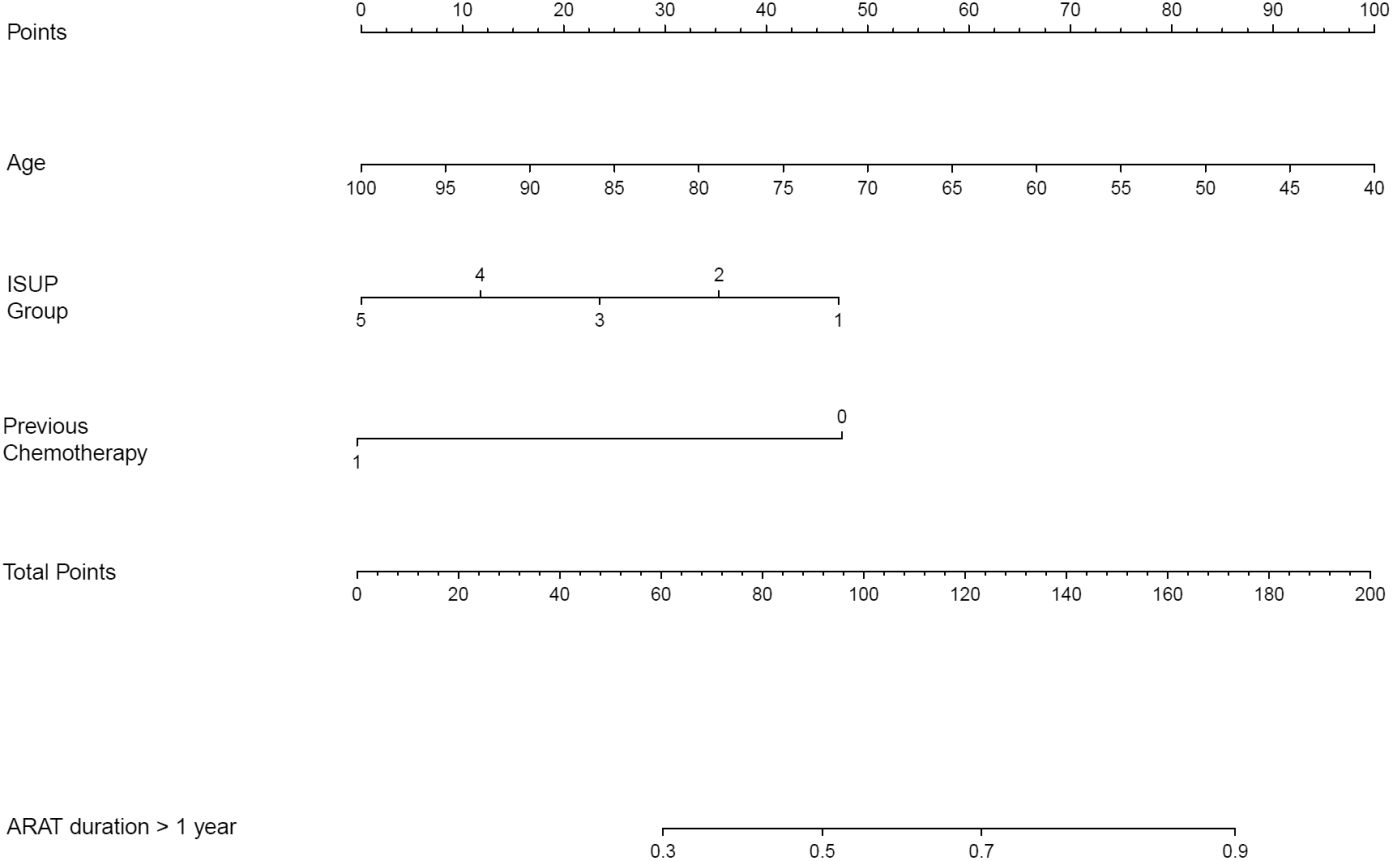
Nomogram to predict the probability of ARAT response duration of more than 1 year.

From our analysis results, the significant predictors of ARAT failure‐free survival were used to establish a prediction model. Nomogram analysis was performed to predict the likelihood of an ARAT duration of > 1 year depending on age, ISUP grading, and chemotherapy‐naïve status. The constructed nomogram is presented in Figure 4.

## 4 Discussion

Although most patients with metastatic prostate cancer initially respond to ADT, they subsequently develop resistance to ADT within 13–16 months [28]. In the previous decades, various new drugs were discovered and have been verified to provide survival‐prolonging benefits for patients with mCRPC. Given the availability of multiple treatment options, the combination or sequence of treatments has become a crucial clinical consideration. To meet various needs, a reliable marker is required to predict the treatment outcome of these new drugs in patients with mCRPC [29, 30, 31]. Detecting early or primary resistance can assist clinicians in selecting a suitable first‐line treatment or making the decision to transition early to the next‐line treatment. This can strengthen the fundamentals of precision medicine, improve therapeutic outcomes, and reduce unnecessary toxicity.

Although abiraterone and enzalutamide can substantially improve OS, approximately 20%–30% of patients still experience primary resistance to abiraterone and enzalutamide [12, 21, 32, 33]. Some studies have focused on predictors for identifying responders and assessing therapeutic responses. Afshar et al. identified several clinical characteristics, including hemoglobin level, ECOG‐PS, and duration of response to ADT, as independent prognostic factors for OS in patients with mCRPC who had a history of chemotherapy [34]. Nadal et al. also reported that when ARAT was implemented as a second‐line therapy, the duration from hormone‐sensitive prostate cancer (HSPC) to CRPC development, PSA response (50% decline) to first‐line novel ARAT, and pretreatment albumin level were significantly correlated with progression‐free survival, whereas performance status, pretreatment albumin level, extent of the disease, and duration from HSPC to CRPC were associated with OS [35]. A recent study also reported that a high GS score was significantly associated with poor OS in patients with mCRPC who underwent ARAT or treatment with docetaxel [35].

In the present study, we performed Cox regression analysis to identify significant predictors of ARAT failure‐free survival and OS in patients with mCRPC. The analysis revealed that patients who were older, had a higher ISUP grade, and had a history of chemotherapy were significantly more likely to experience early ARAT failure. Although several studies have suggested that the duration from HSPC to CRPC development is a significant predictor of the response to ARAT in patients with mCRPC, we did not identify any significant correlation between ARAT failure‐free survival and the duration from HSPC to CRPC development. The nadir PSA level during ADT was identified as a significant predictor of ARAT failure‐free survival in our univariate analysis, but not in our multivariate analysis. The association between the initial response to ADT in patients with mHSPC and the response to ARAT in patients with mCRPC requires further clarification.

In addition to ARAT failure‐free survival, age, ISUP grading, de novo metastasis, duration from HSPC to CRPC development, and nadir PSA during ADT were identified as significant predictors of OS.

On the basis of our multivariate analysis results, we further generated a nomogram to predict the likelihood of ARAT failure‐free survival stratified by age, ISUP grading, and history of chemotherapy. The nomogram can help clinicians to predict the timing of early ARAT failure in patients with mCRPC. When the expected ARAT response duration is short, next‐line therapy such as chemotherapy, PARP inhibitors, immunotherapy, and radiopharmaceutical therapy should be considered first. At this stage, various precision‐medicine screening tools should be used, such as genome testing, immunohistochemical staining of cancer tissue, and radiographic scans (e.g., PSMA or bone scans). These screening tests can help clinicians to make informed decisions regarding the optimal selection of next‐line therapeutic agents.

Among the 319 patients (i.e., patients who underwent ARAT after progression to mCRPC) included in the present study, 193 (60.5%) underwent at least one other life‐prolonging treatment for mCRPC, including rechallenge with another ARAT, chemotherapy, or radium‐223 treatment. This ratio is higher than the real‐world ratio reported in the United States, which is 49% [36]. The key reason for this difference may be the coverage provided by Taiwan's National Health Insurance program, which allows patients with mCRPC to be reimbursed for all three of these treatment agents. Because medical cost is not a barrier to treatment, patients and clinicians can attempt multiple lines of treatment to maximize the duration of disease control.

The therapeutic effects of abiraterone and enzalutamide were compared in the present study. Our univariate analysis indicated that the ARAT failure‐free survival of the patients who took enzalutamide was significantly higher than that of the patients who took abiraterone (18.1 vs. 14.0 months; *p* = 0.017). However, this finding became nonsignificant in our multivariate analysis. This result may be related to the retrospective design and selection bias of our study. In Taiwan, abiraterone was approved earlier than enzalutamide for patients with mCRPC. Consequently, the percentage of patients with a history of chemotherapy was higher among those who took abiraterone than among those who took enzalutamide, and this difference led to the poorer ARAT failure‐free survival outcome in the abiraterone group. For OS, no statistical significance was identified (OS, abiraterone vs. enzalutamide, 42.4 vs. 52.4 months; *p* = 0.053). Notably, these OS values are higher than those reported in other clinical trials; specifically, the PREVAIL trial reported an OS of 35.5 months for enzalutamide, and the COU‐AA‐302 trial reported an OS of 34.7 months for abiraterone. Ethnic differences may explain the more favorable survival outcome of our study relative to those of other studies. Specifically, a study reported that an Asian population exhibited a stronger response to ADT and longer survival times relative to other ethnic groups [37].

In addition to clinical parameters, recent studies have focused on the predictive value of various –omics and biomarker data, including information on genes, transcriptomes, and protein/lipid products. Tumorigenesis and clonal heterogeneity, which are complex processes, and rapid clonal evolutional processes affect not only the natural course of cancer progression but also the response of patients to treatment [38, 39, 40]. Relative to clinical parameters alone, bioinformatics and –omics data can serve as more individualized and precise predictive biomarkers of mCRPC prognosis. For patients with mCRPC, multi‐omics data and clinical parameters can be integrated to improve outcome predictions and responses to specific treatment modalities. To address unmet clinical needs, validating robust clinical parameters and establishing effective multi‐omics clinical models are necessary.

The main limitation of the present study is selection bias due to its retrospective design. Clinicians sequence therapeutic agents and schedule various treatments on the basis of clinical scenarios. Different combinations of treatments have different effects on prognoses and treatment responses. Although we performed various statistical analyses, including multivariate analysis, to eliminate bias, a comparison of randomized trials is still required. Furthermore, all the patients included in the present study underwent ARAT for mCRPC; this differs from current real‐world practice, with most guidelines suggesting the use of ARAT only in an mHSPC setting along with ADT. However, the results of the present study are still useful for patients who underwent ARAT until the mCRPC stage.

## 5 Conclusion

For patients with mCRPC, their response to first‐line ARATs can be predicted by conducting nomogram analysis on the basis of age, ISUP grading, and chemotherapy‐naïve status. For patients with a predicted ARAT response duration of < 1 year, other therapeutic agents should be prioritized. Notably, age, ISUP grading, and nadir PSA during ADT are independent predictors of OS.

## Author Contributions

**I‐Hung Shao:** conceptualization (equal), data curation (equal), writing – original draft (equal), writing – review and editing (equal). **Hsiang‐Shen Wang:** data curation (equal), resources (equal). **Chin‐Hsuan Hsieh:** data curation (equal), supervision (equal). **Tsung‐Lin Lee:** investigation (equal), resources (equal). **Ying‐Hsu Chang:** investigation (equal). **Liang‐Kang Huang:** validation (equal). **Yuan‐Cheng Chu:** investigation (equal). **Hung‐Chen Kan:** data curation (equal), formal analysis (equal). **Po‐Hung Lin:** resources (equal), validation (equal). **Kai‐Jie Yu:** investigation (equal), visualization (equal). **Chun‐Te Wu:** supervision (equal). **Cheng‐Keng Chuang:** funding acquisition (equal). **See‐Tong Pang:** conceptualization (equal), funding acquisition (equal), resources (equal), supervision (equal).

## Ethics Statement

This study has been conducted in accordance with the ethical principles mentioned in the Declaration of Helsinki (2013). This study was approved by Chang Gung Medical Foundation Institutional Review Board. (IRB Number: 202101497A0C602, 201801395B0C503).

## Consent

The authors have nothing to report.

## Conflicts of Interest

The authors declare no conflicts of interest.

## Data Availability

The data that support the findings of this study are available upon request from the corresponding author, upon reasonable request.
